# A retrospective study of the relationship between postoperative urine output and one year transplanted kidney function

**DOI:** 10.1186/s12871-019-0904-6

**Published:** 2019-12-17

**Authors:** Joungmin Kim, Taehee Pyeon, Jeong Il Choi, Jeong Hyeon Kang, Seung Won Song, Hong-Beom Bae, Seongtae Jeong

**Affiliations:** Department of Anesthesiology and Pain Medicine, Chonnam National University Medical School; Chonnam National University Hospital, 42 Jebong-ro Dong-gu, Gwangju, 61469 South Korea

**Keywords:** Kidney transplantation, Urine output, Graft survival, Glomerular filtration rate

## Abstract

**Background:**

Kidney transplantation (KT) is the most obvious method of treating a patient with end-stage renal disease. In the early stages of KT, urine production is considered a marker of successful reperfusion of the kidney after anastomosis. However, there is no clear conclusion about the relationship between initial urine output after KT and 1-year renal function. Thus, we investigated the factors that affect 1-year kidney function after KT, including urine output.

**Methods:**

This retrospective study investigated the relationship between urine output in the 3 days after KT and transplanted kidney prognosis after 1-year. In total, 291 patients (129 living-donor and 162 deceased-donor transplant recipients) were analyzed; 24-h urine volume per body weight (in kilograms) was measured for 3 days postoperatively. The estimated glomerular filtration rate (eGFR), determined by the Modification of Diet in Renal Disease algorithm, was used as an index of renal function. Patients were grouped according to eGFR at 1-year after KT: a good residual function group, eGFR ≥60, and a poor residual function group, eGFR < 60.

**Result:**

Recipients’ factors affecting 1-year eGFR include height (*P* = 0.03), weight (*P* = 0.00), and body mass index (*P* = 0.00). Donor factors affecting 1-year eGFR include age (*P* = 0.00) and number of human leukocyte antigen (HLA) mismatches (*P* = 0.00). The urine output for 3 days after KT (postoperative day 1; 2 and 3) was associated with 1-year eGFR in deceased-donor (*P* = 0.00; *P* = 0.00 and *P* = 0.01). And, postoperative urine output was associated with the occurrence of delayed graft function (area under curve (AUC) = 0.913; AUC = 0.984 and AUC = 0.944).

**Conclusion:**

Although postoperative urine output alone is not enough to predict 1-year GFR, the incidence of delayed graft function can be predicted. Also, the appropriate urine output after KT may differ depending on the type of the transplanted kidney.

**Trial registration:**

Clinical Research Information Service of the Korea National Institute of Health in the Republic of Korea (KCT0003571).

## Background

The incidence of end-stage renal disease (ESRD) is increasing with the prolonged life span and raised prevalence of chronic diseases, such as diabetes and hypertension [1]. Kidney transplantation (KT) is a proven approach to improve quality of life and prolong life expectancy in patients with ESRD [2]. Therefore, it has become important to maintain the function of the transplanted kidney due to the imbalance caused by limited supply and increasing demand. During KT, recipients have been given mannitol [3], dopamine [4], furosemide [5], and fluid loading [6] to enhance transplanted-kidney function. A few of these measures have increased the long-term survival rate of transplanted organs.

Early detection and prevention of renal functional decline are important for the maintenance of normal graft function. Several tests, including urine-based measurements, can be used to evaluate the function of the kidneys. Urine tests are suitable for clinical applications because the necessary samples are easy to obtain. They involve the measurement of specific substances in the urine, such as kidney injury molecule-1 [7], or measurement of the urine volume. In early stages of KT, urine production is considered to be a sign of successful reperfusion after anastomosis [8, 9]. The use of perioperative diuretics to increase urine volume differs among centers with respect to type and dose [10, 11]. Some reports have shown that long-term prognosis can be predicted by urine volume after KT [12, 13]. However, excessive diuresis may occasionally result in a lack of circulating plasma volume or electrolyte imbalances [14].

In general, it is known that the glomerular filtration rate (GFR) is an excellent indicator of overall kidney function [15]. GFR measurements using exogenous markers such as inulin clearance are known to be the most accurate methods. However, use of these markers is laborious and expensive, and thus is rare in clinical practice. Conversely, although there is some inaccuracy, endogenous markers such as serum creatinine (Cr) or cystatin C are used to assess kidney function. Limiting factors for using Cr as a marker of GFR include weight, age, sex and race. The Modification of Diet in Renal Disease (MDRD) equation for GFR estimation was derived from 1628 patients with chronic kidney disease (mean GFR, 40 mL / min / 1.73 m^2^) to overcome some limitations [16].

Transplanted kidneys are known to produce large amounts of urine in the initial stage after transplantation [17]. No criterion for the appropriate urine volume after KT has been established. We aimed to compare urine volume in the 3 days after surgery and the estimated glomerular filtration rate (eGFR) at 1 year postoperatively in patients who received KT.

## Methods

### Study design and ethical statement

This single-center retrospective cohort study was conducted using the electronic medical records of Chonnam National University Hospital. This registry retrospectively collects data regarding recipients’ characteristics and outcomes. Adult patients (age ≥ 20 years) who underwent KT in our center during the 10-year period between 1 January 2008 and 31 December 2017 were included.

The institutional review board of Chonnam National University Hospital approved the study protocol (CNUH-2019-018), and the study was registered with the Clinical Research Information Service of the Korea National Institute of Health in the Republic of Korea (KCT0003571), which belongs to the World Health Organization Registry Network.

### Data collection

In total, 303 kidney transplants, including re-transplantations, were performed during the study period. Patients were excluded from the analysis if they lacked medical records for the first year after surgery, due to death or loss to follow-up; patients aged < 20 years were also excluded. The preoperative information collected was age, sex, height, weight, body mass index (BMI), duration of dialysis, method of dialysis, eGFR, diabetes mellitus (DM) status, hypertension (HTN) status, hepatitis B virus status, hepatitis C virus status, and type of donated kidney. eGFRs were estimated using the MDRD equation. In addition, the characteristics of donated kidney such as age, Creatinine, DM, HTN, and human leukocyte antigen (HLA) mismatch, incidence of delayed graft function (DGF) were also investigated. DGF was defined as hemodialysis performed within 1 week after surgery. Nephropathy was defined as primary kidney disorder, such as IgA nephropathy or autosomal polycystic kidney, but not secondary kidney disorder due to HTN or DM. Our hospital usually measures the amount of urine for 24 h during the 3 days after KT; those data were used in the present study. The total volume (in milliliters) of urine collected over 24 h was divided by the body weight (in kilograms) of the patient. Follow-up data (e.g., eGFR, graft rejection, and viral infection) were collected at 1 year after KT. Graft rejection episodes were defined as biopsy-proven rejection or clinically suspected acute rejection that was improved by empirical steroid pulse therapy. Patients were considered to be positive for viral infection when cytomegalovirus (CMV), Epstein–Barr virus (EBV), or BK polyomavirus (BKV) was detected after KT.

### Outcomes

Chronic kidney disease (CKD) is defined as GFR < 60 mL/min/1.73 m^2^ for 3 months or more [18]. This criterion can be applied to KT patients [19] and, the degree of GFR impairment at 1 year post-KT has prognostic value and is associated with lower GFR at 5 years, higher risk of eventual graft failure, and cardiovascular death [20]. The patients were grouped according to eGFR at 1 year after KT: a good residual function group, eGFR ≥60, and a poor residual function group, eGFR < 60. The primary goal was to assess the relationship between the eGFR at 1 year postoperatively and urine output during the 3 days after KT. The secondary goal was to assess the association of eGFR at 1 year after KT with other patient data, such as donated kidney’s demographics, fluid and diuretic dose, rejection and infection.

### Statistical analysis

For demographic data, the independent two sample t-test for normal continuous data or the Wilcoxon rank-sum test for non-normal continuous data were used as appropriate. The normality was verified by Shapiro-Wilk test. The categorical data was analyzed by the Chi-squared test or Fisher’s Exact test. A subgroup analysis according to the type of transplanted kidney was performed. A bonferroni correction was used to adjust type I error for multiple comparisons in subgroup analysis and *P* values <.025 was considered as statistically significant. The analysis of usefulness of postoperative urine output for the prediction of delayed graft function and residual renal function at 1 year after transplantation used receiver-operating-characteristic (ROC) curve techniques. The statistical analysis was performed using R version 3.6.0 (The R Foundation for Statistical Computing, Vienna, Austria).

## Results

**Fig. 1:**
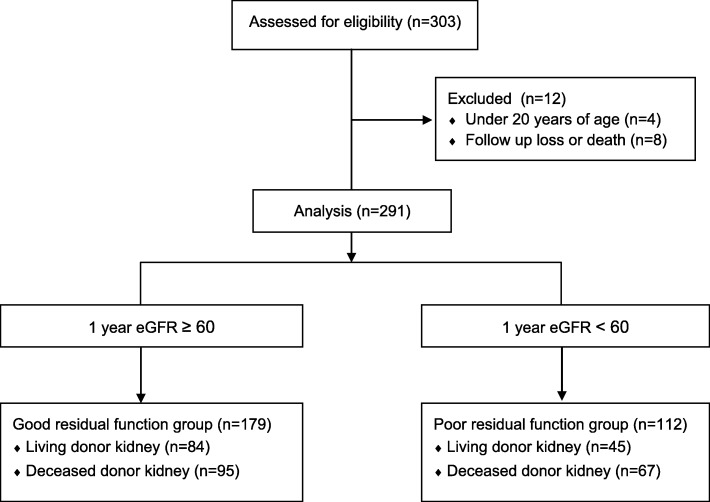
Data collection

During the study period, 303 kidney transplants were performed. Four patients aged < 20 years were excluded. Eight patients who were lost to follow-up or who died within 1 year after KT were also excluded. Finally, a total of 291 patients were included in the analysis (Fig. 1).

### Baseline characteristics of the study population

Demographic data of KT recipients were not statistically different between two groups except height, weight and BMI (Table 1). Age and HLA mismatch were statistically significant between the two groups in the donated kidney’s demographic data (Table 2).

**Table 1:** Demographic data of kidney recipients

	Good residual function group (*n* = 179)	Poor residual function group (*n* = 112)	*P* value
Age	47.0 [38.0;54.5]	47.0 [38.5;55.0]	.84
Gender (male/female)	111 (62.0%) / 68 (38.0%)	79 (70.5%) / 33 (29.5%)	.18
Height (cm)	164.0 [158.0;171.0]	167.5 [160.0;172.0]	.03
Weight (kg)	60.5 ± 9.7	65.2 ± 12.2	.00
BMI	22.3 ± 3.0	23.4 ± 3.4	.00
Pre-transplant dialysis duration (month)	36.0 [5.0;72.0]	48.0 [12.0;72.0]	.12
Pre-transplant dialysis type			.53
HD	108 (60.3%)	66 (58.9%)	
PD	44 (24.6%)	33 (29.5%)	
HD + PD	10 (5.6%)	7 (6.2%)	
None	17 (9.5%)	6 (5.4%)	
Pre-operative eGFR (ml/min/1.73m^2^)	5.5 [4.3; 7.6]	5.3 [4.0; 7.0]	.15
KT history			.59
1st	167 (93.3%)	107 (95.5%)	
2nd	12 (6.7%)	5 (4.5%)	
ABO incompatibility			.43
Matched	170 (95.0%)	103 (92.0%)	
Un-matched	9 (5.0%)	9 (8.0%)	
Donated Kidney			.31
Living	84 (46.9%)	45 (40.2%)	
Deceased	95 (53.1%)	67 (59.8%)	
HBV positive	15 (8.4%)	13 (11.6%)	.48
HCV positive	3 (1.7%)	2 (1.8%)	1.00
Nephropathy positive	10 (5.6%)	9 (8%)	.56
HTN positive	155 (86.6%)	103 (92.0%)	.22
DM positive	44 (24.6%)	30 (26.8%)	.77

Data are present as mean ± standard deviation or median [95% confidence interval] or number (%)

Good residual function group: 1 year eGFR ≥60, Poor residual function group: 1 year eGFR < 60

*BMI* Body mass index, *HD* Hemodialysis, *PD* Peritoneal dialysis, *eGFR* Estimated glomerular filtration rate, *KT* Kidney transplantation, *HBV* Hepatitis B virus, *HCV* Hepatitis C virus, *HTN* Hypertension, *DM* Diabetes mellitus

**Table 2:** Donated kidney characteristics

	Good residual function group	Poor residual function group	*P* value
All			
	(*n* = 179)	(*n* = 112)	
Age	40.0 [27.5;49.0]	50.0 [44.0;56.0]	.00
Creatinine (mg/dl)	0.9 [0.7; 1.2]	0.9 [0.7; 1.2]	.45
DM positive	5 (2.8%)	9 (8.0%)	.08
HTN positive	210.1 ± 94.3	176.9 ± 94.5	.04
HLA mismatching	3.0 [2.5; 4.0]	4.0 [3.0; 5.0]	.00
DGF positive	4 (2.2%)	4 (3.6%)	.75
Living donor kidney			
	(*n* = 84)	(*n* = 45)	
Age	40.2 ± 11.7	48.4 ± 9.2	.00
Creatinine (mg/dl)	0.8 [0.6; 0.9]	0.8 [0.6; 0.9]	.80
DM positive	0 (0.0%)	1 (2.2%)	.75
HTN positive	7 (8.3%)	3 (6.7%)	1.00
HLA mismatching	3.0 [2.0; 3.0]	3.0 [3.0; 4.0]	.02
DGF positive	2 (2.4%)	0 (0.0%)	.76
Deceased donor kidney			
	(*n* = 95)	(*n* = 67)	
Age	39.0 [24.0;50.5]	49.5 [44.0;56.0]	.00
Creatinine (mg/dl)	1.0 [0.7; 1.5]	1.1 [0.8; 1.4]	.65
DM positive	5 (5.3%)	8 (11.9%)	.21
HTN positive	12 (12.6%)	13 (19.4%)	.34
HLA mismatching	3.0 [3.0; 4.0]	4.0 [3.0; 5.0]	.02
DGF positive	2 (2.1%)	4 (6.0%)	.39

Data are present as mean ± standard deviation or median [95% confidence interval]

*HTN* Hypertension, *DM* Diabetes mellitus, *HLA* Human leukocyte antigen

### Comparison with 1 year eGFR and postoperative urine output, amounts of fluid and diuretics

The postoperative urine output and the overall 1 year prognosis of the transplanted kidney were related to postoperative 3 days in all patients (Table 3). Interestingly, in the subgroup analysis, urine output and residual kidney function was not associated with a living-donor kidney group, whereas in patients who received a deceased-donor kidney group showed association was evident on all 3 days postoperatively.

**Table 3:** Relationship of 1 yr residual kidney function and postoperative urine output (ml/kg/hr) of 1st, 2nd, and 3rd day

	Good residual function group	Poor residual function group	*P* value
All			
	(*n* = 179)	(*n* = 112)	
POD1	13.2 [10.0;17.8]	9.5 [5.8;15.2]	.00
POD2	8.2 [6.1;10.7]	7.1 [5.1; 9.3]	.00
POD3	6.9 [5.2; 8.8]	6.1 [4.2; 7.9]	.01
Living donor kidney			
	(*n* = 84)	(*n* = 45)	
POD1	16.8 [12.9;20.4]	14.8 [11.4;18.8]	.06
POD2	8.0 [6.3;10.3]	8.0 [6.0; 9.1]	.42
POD3	6.8 [5.1; 8.8]	6.8 [5.7; 7.8]	.61
Deceased donor kidney			
	(*n* = 95)	(*n* = 67)	
POD1	11.1 [7.1;14.5]	7.2 [4.5;10.1]	.00
POD2	8.3 [5.9;11.2]	6.9 [4.1; 9.3]	.00
POD3	6.9 [5.2; 9.0]	5.7 [3.4; 8.1]	.01

Data are present as median [95% confidence interval]

Good residual function group: 1 year eGFR ≥60, Poor residual function group: 1 year eGFR < 60

*POD* Postoperative day

Amounts of fluid administration for 3 days after surgery were compared (Table 4). The amount of fluid administered was larger in the good residual function group. This difference is thought to be due to postoperative fluid therapy in our institution. Patients receive 15 ml/kg for 1 h immediately after surgery and an additional dose of fluids given as same volume of urine output for 3 days. The basic maintenance of fluid was 1 ml/kg/hr. and the amount was reduced as the diet progressed. All of the administered fluids were crystalloids such as normal saline or Hartman solution, and the choice of fluid was determined by the electrolyte concentration of the patient.

**Table 4:** Relationship of residual kidney function and postoperative fluid therapy (ml/kg/hr) of 1st, 2nd, and 3rd day

	Good residual function group	Poor residual function group	*P* value
All			
	(*n* = 179)	(*n* = 112)	
POD1	13.9 [9.9;19.1]	11.2 [7.5;16.6]	.00
POD2	11.2 [7.8;13.4]	8.6 [6.1;12.1]	.00
POD3	9.1 [6.3;11.3]	7.6 [4.9;10.3]	.00
Living donor kidney			
	(*n* = 84)	(*n* = 45)	
POD1	17.0 [13.0;21.3]	16.2 [13.4;20.7]	.30
POD2	11.6 [9.1;13.9]	10.3 [9.0;13.8]	.37
POD3	9.1 [7.0;10.9]	8.2 [6.4;10.0]	.09
Deceased donor kidney			
	(*n* = 95)	(*n* = 67)	
POD1	11.2 [7.4;15.1]	9.0 [6.3;11.9]	.02
POD2	10.6 [7.0;13.3]	7.1 [5.2; 9.8]	.00
POD3	8.9 [5.7;11.8]	7.0 [4.3;10.4]	.03

Data are present as median [95% confidence interval]

Good residual function group: 1 year eGFR ≥60, Poor residual function group: 1 year eGFR < 60

*POD* Postoperative day

There was no statistical difference between the two groups when comparing the amount of diuretics (furosemide, mannitol) administered for 3 days and 1 year eGFR (Tables 5 and 6).

**Table 5:** Relationship of residual kidney function and postoperative furosemide dosage (mg) of 1st, 2nd, and 3rd day

	Good residual function group	Poor residual function group	*P* value
All			
	(*n* = 179)	(*n* = 112)	
POD1	40 [20; 80]	40 [20; 80]	.61
POD2	40 [20; 80]	40 [20; 60]	.31
POD3	20 [20; 40]	20 [20; 40]	.08
Living donor kidney			
	(*n* = 84)	(*n* = 45)	
POD1	40 [20; 80]	40 [20; 80]	.69
POD2	60 [40; 100]	60 [40; 80]	.56
POD3	20 [20; 40]	20 [20; 40]	.21
Deceased donor kidney			
	(*n* = 95)	(*n* = 67)	
POD1	60 [20; 80]	40 [20; 60]	.17
POD2	20 [20; 60]	20 [20; 60]	.68
POD3	20 [20; 40]	20 [20; 40]	.20

Data are present as median [95% confidence interval]

Good residual function group: 1 year eGFR ≥60, Poor residual function group: 1 year eGFR < 60

*POD* Postoperative day

**Table 6:** Relationship of residual kidney function and postoperative mannitol dosage (g) of 1st, 2nd, and 3rd day

	Good residual function group	Poor residual function group	*P* value
All			
	(*n* = 179)	(*n* = 112)	
POD1	30 [30; 30]	30 [30; 30]	.51
POD2	30 [30; 30]	30 [30; 30]	.39
POD3	0 [0; 30]	30 [0; 30]	.28
Living donor kidney			
	(*n* = 84)	(*n* = 45)	
POD1	30 [30; 30]	30 [0; 30]	.65
POD2	30 [30; 30]	30 [30; 30]	.35
POD3	0 [0; 30]	0 [0; 30]	.85
Deceased donor kidney			
	(*n* = 95)	(*n* = 67)	
POD1	30 [30; 30]	30 [30; 30]	.49
POD2	30 [30; 30]	30 [30; 30]	.83
POD3	30 [0; 30]	30 [0; 30]	.44

Data are present as median [95% confidence interval]

Good residual function group: 1 year eGFR ≥60, Poor residual function group: 1 year eGFR < 60

*POD* Postoperative day

### Prediction of DGF and 1 year residual graft function through postoperative urine output

Postoperative day 2 urine output was the highest in the correlation between 3 days postoperative urine output and DGF development (Table 7).

**Table 7:** Predicting DGF incidence with postoperative urine output using ROC curve

	AUC
POD1UO	0.913
POD2UO	0.984
POD3UO	0.944
POD1UO + 2 + 3	0.969

*AUC* Area under curve, *PODUO* Post-operative day urine output

**Fig. 2:**
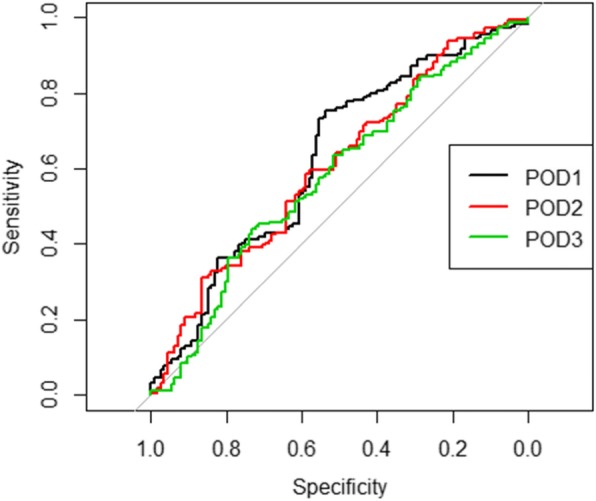
ROC curve of urine output POD1–3 for 1 year eGFR. (POD = postoperative day)

The relationship between postoperative urine output at 3 days and 1 year eGFR was analyzed by ROC curve (Fig. 2).

### Comparison with 1 year eGFR and postoperative rejection, viral infection

Good residual function group was associated with fewer rejection episodes in all patients; subgroup analysis yielded similar findings (Table 8).

**Table 8:** Rejection episode

	Good residual function group	Poor residual function group	*P* value
All			
	(*n* = 179)	(*n* = 112)	.00
Positive	9 (5%)	33 (29%)	
Negative	170 (95%)	79 (71%)	
Living donor kidney			
	(*n* = 84)	(*n* = 45)	.00
Positive	6 (7%)	16 (36%)	
Negative	78 (93%)	29 (64%)	
Deceased donor kidney			
	(*n* = 95)	(*n* = 67)	.00
Positive	3 (3%)	17 (25%)	
Negative	92 (97%)	50 (75%)	

Data are present as number (%)

Good residual function group: 1 year eGFR ≥60, Poor residual function group: 1 year eGFR < 60

Good residual function group was also associated with fewer viral infections in all patients; subgroup analysis yielded similar findings (Table 9). The cohort contained 45 patients with CMV infection, 9 patients with BK infection, 6 patients with CMV and BK co-infection, and no patient with EBV infection (data not shown).

**Table 9:** Viral infection

	Good residual function group	Poor residual function group	*P* value
All			
	(*n* = 179)	(*n* = 112)	.00
Positive	19 (11%)	35 (31%)	
Negative	160 (89%)	77 (69%)	
Living donor kidney			
	(*n* = 84)	(*n* = 45)	.00
Positive	4 (5%)	13 (29%)	
Negative	80 (95%)	32 (71%)	
Deceased donor kidney			
	(*n* = 95)	(*n* = 67)	.01
Positive	15 (16%)	22 (33%)	
Negative	80 (84%)	45 (67%)	

Data are present as number (%)

Good residual function group: 1 year eGFR ≥60, Poor residual function group: 1 year eGFR < 60

## Discussion

Early diuresis after KT is a good indicator of transplanted kidney function [21]. However, the relationship between initial urine output and long-term survival of the transplanted kidney is unclear. Aigner et al. [22] reported that the initial postoperative urine volume was an accurate predictor of delayed graft function (DGF). There are many definitions, but DGF is usually defined as using dialysis the first week after surgery [23]. The long-term prognosis of a transplanted kidney is also related to postoperative urine output [12, 13]. However, Chisholm et al. [24] reported that they found no association between initial diuresis and 12-month graft survival. The present study shows that the 1 year eGFR after KT was associated with urine outputs on postoperative 3 days. However, in subgroup analysis, urine output on all three postoperative days was significantly associated with residual graft function in deceased-donor KT but not in living-donor KT.

In this study, deceased-donor kidney recipient was associated with postoperative urine output and 1-year eGFR. Urine production begins as blood moves to the glomerulus and is filtered out of the glomerular barrier. The renin–angiotensin–aldosterone system (RAAS) regulates renal blood flow and the GFR by modulating resistance of the renal afferent and efferent arterioles. Regardless of the type of fluid, when a sufficient volume of fluid enters the blood vessel, the amounts of renin and aldosterone decrease and urine volume increases [25]. Renin and aldosterone increase due to prolonged cold ischemic time during KT in deceased-donor kidneys. This elevation is maintained until the second postoperative day [26]. The differences in urine volume and graft function between living-donor and deceased-donor kidney recipients in our study may be attributed to activation of the RAAS by prolonged cold ischemic time. Koller et al. [27] reported that 41% of deceased-donor kidney transplants were complicated by DGF. They found that deceased-donor kidneys exhibited higher plasma renin and angiotensin II levels after KT, compared with living-donor kidneys (renin, 5.1 vs. 2.6 ng/mL/h, *P* < .02; angiotensin II, 62.8 vs. 48.5 pg/mL, *P* < .01). In addition, Oberbauer et al. [28] reported that 10-year graft survival rates were 59% in angiotensin-converting-enzyme inhibitor/angiotensin-receptor blocker users and 41% in patients who did not use either type of drug.

Globally, the 1-year graft survival rate of overall KT is > 90%, and this rate is gradually improving for deceased-donor KT [29]. However, the 20-year transplanted kidney survival rate is 21% [30]. The method of raising the long-term survival rate of transplanted kidney is avoidance of known risk factors. Donor risk factors associated with graft failure are age > 60 years, history of HTN, cerebrovascular cause of death, and pre-harvest serum Cr > 150 mol/L [31]. Risk factors measured in this study were age, hypertension and HLA mismatch. Recipient risk factors associated with graft failure include age, increasing plasma renin activity, BMI, prior transplant, dialysis at the time of KT, and hepatitis C virus infection [32]. Our study also showed that recipient height, weight and BMI affect graft function. Navis et al. [33] reported that increased BMI was associated with increased glomerular filtration pressure, which adversely affected long-term graft survival.

In this study, postoperative viral infection was associated with the 1-year eGFR. There may be several types of viral infections after KT, but CMV and BKV are common. Viral infections may affect acute or chronic rejection episodes. Reportedly, 72% of CMV-positive recipients developed rejection, whereas CMV-negative recipients had only a 17% rejection rate [34]. The mechanism of CMV-induced allograft rejection is as follows. First, activation of HLA class I antigen-specific T cells due to cross-reactivity with CMV antigens. Second, release of inflammatory cytokines [eg, IL (interleukin) -1, IL-6, IL-8, and tumor necrosis factor-α] and direct damage of endothelial cells. Third, these complex interactions not only increase the expression of HLA class II molecules in allogeneic grafts, but also produce adhesion molecules of white blood cells and endothelial cells [35].

Finally, the results of this study show a correlation between the occurrence of rejection episodes and the 1-year eGFR. Esteve et al. [36] reported that patients with rejection episodes were at increased risk of late graft failure. In addition, acute rejection episodes affected graft survival, regardless of the time of onset [37]. Immunosuppressant use is inevitable in KT patients to achieve control of rejection, but it renders the recipient susceptible to viral infection and reactivation [38].

In the present study, most KT were carried out by one surgeon and there were few variables due to surgery. However, this study has the following limitations. First, this study was retrospectively conducted in a single institution. Therefore, the sample size was small and there was no direct measurement of renin and aldosteron. However, it is known that renin and aldosterone levels are raised in deceased donor KT. Second, demographic data are different between the groups (for example BMI), so we tried to compensate for this difference by using urine volume per weight rather than post-operative urine output itself. Third, The incidence of DGF in this study was lower than in other studies [39]. This may reveal that postoperative urine output is a risk factor for the development of DGF, but is insufficient to explain the outcome of 1 year eGFR. Fourth, we used eGFR with MDRD to assess kidney function. This formula tends to be somewhat higher than the actual glomerular filtration rate in Asians, including Japan, and suffers from a slight drop in accuracy when glomerular filtration rate is normal or slightly decreased [40]. But, the MDRD equation was the most accurate of the creatinine-based equations [41]. Fifth, we did not analyze post-transplantation blood pressure control statuses of the recipients or the types of immunosuppressive drug used after KT.

## Conclusions

Sufficient urine output after immediate KT reflects proper blood supply to the transplanted kidney and the absence of stenosis or leakage at the anastomosis site. Early postoperative urine output was associated with 1-year postoperative graft function in deceased-donor kidney recipients, but not in living-donor kidney recipients. This difference may be caused by activation of the RAAS due to prolonged cold ischemic time during the preparation of deceased-donor kidneys. Therefore, we recommend that the approach to achieving proper urine volume after KT be adjusted depending on the type of transplanted kidney.

## Data Availability

The datasets generated and analyzed during the current study are available from the corresponding author on reasonable request.

## Abbreviations

BKV: BK poliomavirus
BMI: Body mass index
CMV: Cytomegalovirus
Cr: Creatinine
DGF: Delayed graft function
DM: Diabetes mellitus
EBV: Epstein-Barr virus
eGFR: Estimated glomerular filtration rate
ESRD: End-stage renal disease
HLA: Human leukocyte antigen
HTN: Hypertension
IL: Interleukin
KT: Kidney transplantation
MDRD: Modification of Diet in Renal Disease
POD: Postoperative day
RAAS: Renin-angiotension-aldosterone system
ROC: Receiver-operating-characteristic
